# Planting Sentinel European Trees in Eastern Asia as a Novel Method to Identify Potential Insect Pest Invaders

**DOI:** 10.1371/journal.pone.0120864

**Published:** 2015-05-20

**Authors:** Alain Roques, Jian-ting Fan, Béatrice Courtial, Yan-zhuo Zhang, Annie Yart, Marie-Anne Auger-Rozenberg, Olivier Denux, Marc Kenis, Richard Baker, Jiang-hua Sun

**Affiliations:** 1 INRA UR 633 Zoologie Forestière, Orléans, France; 2 School of Forestry and Bio-technology, Zhejiang Agriculture and Forestry University, Lin'an, China; 3 State key laboratory of Integrated Management of pest Insects and Rodents, Institute of Zoology, Chinese Academy of Sciences, Beijing, China; 4 CABI, Delémont, Switzerland; 5 Department for Environment Food and Rural Affairs, Sand Hutton, York, United Kingdom; Natural Resources Canada, CANADA

## Abstract

Quarantine measures to prevent insect invasions tend to focus on well-known pests but a large proportion of the recent invaders were not known to cause significant damage in their native range, or were not even known to science before their introduction. A novel method is proposed to detect new potential pests of woody plants in their region of origin before they are introduced to a new continent. Since Asia is currently considered to be the main supplier of insect invaders to Europe, sentinel trees were planted in China during 2007-2011 as an early warning tool to identify the potential for additional Asian insect species to colonize European trees. Seedlings (1-1.5 m tall) of five broadleaved (*Quercus petraea*, *Q*. *suber*, *Q*. *ilex*, *Fagus sylvatica*, and *Carpinus betulus*) and two conifer species (*Abies alba* and *Cupressus sempervirens*) were planted in blocks of 100 seedlings at two widely separated sites (one in a nursery near Beijing and the other in a forest environment near Fuyang in eastern China), and then regularly surveyed for colonization by insects. A total of 104 insect species, mostly defoliators, were observed on these new hosts, and at least six species were capable of larval development. Although a number of the insects observed were probably incidental feeders, 38 species had more than five colonization events, mostly infesting *Q*. *petraea*, and could be considered as being capable of switching to European trees if introduced to Europe. Three years was shown to be an appropriate duration for the experiment, since the rate of colonization then tended to plateau. A majority of the identified species appeared to have switched from agricultural crops and fruit trees rather than from forest trees. Although these results are promising, the method is not appropriate for xylophagous pests and other groups developing on larger trees. Apart from the logistical problems, the identification to species level of the specimens collected was a major difficulty. This situation could be improved by the development of molecular databases.

## Introduction

The globalization of economies, expanding world-wide trade, and climate change are all factors that contribute to the accelerated international movement and establishment of alien organisms, allowing them to overcome geographic barriers [1–4]. At present, the number of unintentional introductions, which are typically by-products of economic activity, are much higher than those introduced intentionally, e.g., for forestry, agriculture, hunting, fur farming, or biological control [5]. In Europe, for instance, the arrival of exotic terrestrial arthropods exponentially increased during the second half of the 20^th^ century, mostly because of phytophagous insect species inadvertently transported with their hosts with the trade in plants for planting (defined as plants intended to remain planted, to be planted or replanted) and plant products [6]. Similarly, Liebhold et al. [7] assumed that nearly 70% of damaging exotic forest pests and pathogens established in the US are most likely to have entered with imported live plants. Additional introductions occur when species hitchhike in containers and on commodities that may or may not include their host plants [1, 8–10]. This trend is not predicted to slow down in the near future. Aukema et al. [11] pointed out that insect and pathogen plant pests continue to become established in the United States despite the implementation of regulations designed to reduce the rate of alien introductions.

Although only a small fraction of the introduced species actually establish and become invasive [12], biological invasions have already been shown to impose enormous costs not only to agriculture and forestry but also to threaten human health and biodiversity [13–16]. The best way to reduce the likelihood of exotic species invasions is to prevent their establishment, but this depends on identifying potential invasive species in advance of their potential entry and establishment. However, predicting which of the many potentially invasive species are most likely to establish in a particular region or country presents a significant challenge to researchers as well as to government agencies [17]. For plant pests, this is mainly done through pest risk analyses (PRAs) [18–19]. However, such approaches mean evaluating and prioritizing each species individually across extensive lists of potential invaders. This process is generally based upon expert opinion which is susceptible to context dependence and motivational bias, and thus can lead to misleading prioritizations [20]. More scientifically-based methods have been developed such as the use of climate envelope models [21], mechanistic niche modelling [22] and other statistical methods [23– 24] but the accuracy of the predictions still remains in question [25].

An alternative approach proposed by Worner and Gevrey [26], Paini et al. [27–28] and Worner et al. [29] is to use self- organizing maps (SOM), a form of artificial neural network, to analyze simultaneously large datasets of invasive species through the study of species assemblages; i.e., groupings of species that co-occur in the same place and at the same time. This allows the identification of patterns of association amongst invasive species, whereby regions with similar suites of invasive species are clustered and a region-specific likelihood of establishment index is generated for each species. This is based on the hypothesis that any species that is commonly found within a particular species assemblage is more likely to establish in a region where that species assemblage is found [28]. Paini et al. [28] recently used this method to analyze the worldwide distribution of 486 invasive fungal pathogens and Worner & Gevrey [26] tested it to generate a quantitative estimate of the risk of establishment in New Zealand for a pool of 844 exotic insect pest species included in the CABI Crop Protection Compendium [30]. Using SOM analysis to prioritize the biosecurity risks to Australia of plant-parasitic nematodes allowed Singh et al. [31] to confirm the risks from frequently quarantined pests but also to identify additional species that could be new threats. In the same way, Eschen et al. [32] tried to predict the likelihood of establishment of tree pests using hierarchical cluster analysis based on distribution data for 1009 pests in 344 regions. They pointed out that the quality of the assessment largely depends on the quality of pest distribution data. This appeared to be highly variable between countries and organism groups, and often based on historical records which do not take into account the most recent trends in trade, e.g. the significant increase in the import of live plant material into the EU from China in recent years [32].

An intrinsic weakness in all these methods is the reliance on only considering the plant pests observed in the countries of origin or the invasive species that have already established in the countries of introduction. Liebhold et al. [7] pointed out that one widely respected and outspoken criticism is that pests that coevolved with their hosts in the country of origin are unlikely to be sufficiently damaging to allow experts to predict the risk they pose to novel ecosystems and hosts. In addition, introduced species are usually released from their natural enemies which may have restricted them to such low population densities in their area of origin that they are not considered as pests [33]. Indeed, a large proportion of the recent insect invaders throughout the world were not known to cause significant damage in their country of origin, nor could they be identified in any invasive pest assemblage before their first arrival outside their native range. A notorious example is the emerald ash borer (*Agrilus planipennis*) that is relatively harmless to indigenous ash trees in its native Far-Eastern Asia [34] but is now one of the most important forest pests in North America [35]. The issue is even more critical for pathogens with many of the most serious forest pathogens, such as *Phytophthora ramorum* that causes ramorum blight, not even being known before they were introduced into a new continent, [36].

Thus, growing plants in continents where they are non-native and studying their subsequent colonization by indigenous insects and pathogens enables potential invaders, even those that are not known anywhere else as pests, to be detected before they are introduced into a new continent. In 2002, the US National Research Council already recommended that an international plant sentinel network should monitor plants native to the United States that are growing in other countries in order to determine the species to which they are susceptible and to evaluate the potential for these species to arrive in the United States [37]. Botanical gardens and arboreta effectively constitute excellent tools because they exist all over the world and, by collecting and planting large numbers of introduced and native plant species together, they make it possible to survey for possible shifts of phytophagous species from the natives to the exotics [38– 39]. However, these collections often include only a few specimens of each plant species, with a limited genetic variability, and are thus not suitable for statistical analyses. Another method consists of deliberately growing sentinel plantations of selected species in other continents, and then studying the indigenous pests that damage the plants.

Our experiment was therefore designed to test the advantages and drawbacks of such plantations as a novel method for the early detection of potential invertebrate and fungal pathogen invaders. Given that the majority of the exotic plant pests that have recently established in Europe are of Asian origin [6], especially those related to woody plants [40], and the trade with Eastern Asia is rapidly increasing, we decided to set up sentinel plantations of European trees in China. We monitored the development of damage caused by native Chinese organisms in order to provide an early warning of the Asian pest species that could potentially affect European trees in the near future. Because the majority of the exotic insects related to woody plants apparently arrived in Europe through the ‘plants for planting’ pathway [41], one of the plantations was established in a nursery area whereas the other was planted in a forested zone. This paper provides the results concerning the colonization of the European trees by Chinese insects. Another paper will present the pathogen data (Vettraino et al., in prep.).

## Materials and Methods

### Establishment of the sentinel plots

Seven species of widely distributed Mediterranean European and temperate trees were used for sentinel plantings in China. These species have been previously exported, unfrequently to China. They consisted of five broadleaved species, four in the family Fagaceae (three oaks: *Quercus petraea* [Mattuschka] Liebl., *Q*. *suber* L., and *Q*. *ilex* L and European beech, *Fagus sylvatica* L.) and one in the family Corylaceae (hornbeam, *Carpinus betulus* L.). Two conifer species were also included: silver fir (*Abies alba*, Miller of the family Pinaceae) and evergreen cypress (*Cupressus sempervirens* L. of the family Cupressaceae). No pines (*Pinus* spp.) could be planted because they are prohibited under the Chinese Animal and Plant Quarantine and Inspection Regulations. Two sentinel tree trials were established at locations ca. 1200 km apart. In order to study the possible association with the plants for planting pathway, the first plot was set up in a suburban nursery area in Xiaotangshan town, Changping district (40.146239N; 116.451817E; 37m elevation), approximately 20 km northeast of central Beijing. A number of native conifer (*Pinus*, *Juniperus*, *Thuja*) and broadleaved tree species such as *Ailanthus*, *Tilia*, *Robinia*, *Populus* and *Salix* are grown in surrounding nurseries. The second plot was established near Fuyang (30,003333 N; 119,799722 E; 110 m elevation), ca. 40 km southwest of Hangzhou (Zhejiang province, Eastern China), in a small farm completely surrounded by a mixed conifer and broadleaved forest composed of *Pinus massoniana*, *Cunninghamia lanceolata*, various species of Fagaceae and bamboos. The surrounding environment also included field crops, especially rice. The seedlings were planted at both locations with the agreement of the land owners. No more specific permissions were required as these field studies did not involve endangered or protected species.

The Beijing site was planted in May 2007 with four of the European species: *Quercus suber*, *Q*. *ilex*, *Abies alba* and *Cupressus sempervirens*. The Fuyang site was planted in May 2008 with the seven species listed above. A total of 100 seedlings were planted per tree species and per site making a total of 1100 seedlings. The seedlings originated from French commercial nurseries. Before export to China, they were individually submitted to thorough phytosanitary inspections by quarantine services to ensure the absence of any insect or pathogen damage, and an additional insecticide and fungicide treatment was applied. The seedlings were exported bare-rooted except for the conifers whose roots were coated with perlite, this growing medium having been checked beforehand and authorized by the National Quarantine Bureau of China. When they arrived in China, they were held for inspection at a quarantine facility in Shanghai for nearly 4 weeks before their release was permitted. The seedlings were immediately planted after being allowed to enter China. The plantings were carried out by students of the Institute of Zoology of the Chinese Academy of Sciences at Beijing and by local farmers at Fuyang. At the latter site, the soil had been treated with insecticide prior to plantation in order to eliminate insect larvae. The initial height of the seedlings was ca. 1–1.5 m with a trunk diameter of 1.0–1.5 cm at the soil level, depending on the tree species. At each site, they were planted at a distance of 50 cm from each other in 4 blocks of 25 seedlings per species. The location of each block in the plot was assigned so that two blocks of the same tree species were not contiguous. The seedlings were watered when planted, but no additional treatments (e.g., fertilization) were applied. All the seedlings were individually identified with waterproof plastic tags.

### Survey of the colonization of European trees by Chinese insects

The Beijing trial was visited every month from May to October from 2007 to 2011, and at Fuyang every two weeks during the same months from 2008 to 2011. Each visit consisted of an individual examination of all seedlings for the presence of phytophagous insects and/or damage. Each seedling was then beaten over a collecting sheet in order to detach and collect the organisms present. The total numbers of insects collected per tree species were then compared between the two plots using a Student’s t test after assessment of the normality of the distribution (Statistix 9.0).

A new damage morphotype was defined whenever a new type of damage was observed on a leaf, bud, branch or stem, and recorded in a photograph. The percentage of leaves affected per morphotype was assessed for each seedling as well as the total foliar damage considering that a leaf could present multiple damage morphotypes. The amount of damage on the other organs of the seedlings was simply counted. Any seedlings found dead were systematically removed and the roots examined for the presence of insect larvae or other organisms. The percentage of dead seedlings per block and the total foliar damage was then compared between tree species within each plot. Because these variables did not follow a normal distribution (as shown by the Kolmogorov–Smirnov test), the comparisons were computed using a non-parametric Kruskall-Wallis test followed by an all-pairwise comparaisons of mean ranks at α = 0.05 (Statistix 9.0).

Damage morphotypes were tentatively attributed to insect species by both visual observation and rearing. When adult insects were observed on seedlings, the apparent damage caused was recorded. These insects were then stored in 95°alcohol for further genetic analyses. The larvae found on seedlings were divided into two lots whenever there were more than two individuals of the same morphological characteristics. Larvae in the first lot were tentatively reared to adults *in situ* by enclosing them individually into a gauze bag together with the affected part of the plant. Their development status and the subsequent damage (number of attacked leaves, damage patterns) was recorded every 15 days. The other half of the individuals was preserved in 95° alcohol.

At the end of the experiment in autumn 2011, all the surviving seedlings at both sites were dug up and burnt because the local owners were reluctant to keep these European trees on their land.

### Insect identification

The adults collected were sent for morphological identification to Chinese taxonomists, and if necessary to specialists elsewhere. Since in most cases it was impossible to identify the larvae collected due to the absence of taxonomic keys, they were first assigned to morphospecies and then systematically subjected to molecular analyses using the mtDNA barcode gene in order to compare them with the existing barcode datasets.

Whole genomic DNA was extracted from individual specimens using a Nucleospin Tissue XS Kit (Macherey-Nagel, Düren, Germany). Identification was done by amplifying a 658 bp fragment of the mitochondrial gene (mtDNA) cytochrome oxidase c subunit 1 (COI) with the polymerase chain reaction (PCR). Reactions were performed in 25 μl volumes containing 2 μl of DNA template (concentration around 50ng/μl), 1x PCR Buffer with 1,5mM MgCl_2_ (Qiagen, Hilden, Germany), 0,5 mM additional MgCl_2_, 100 μM of each dNTP, 2,5 U Taq DNA polymerase from Taq PCR Core Kit (Qiagen, Hilden, Germany), and 200 nM each of the primers LCO1490 (5’-GGTCAACAAATCATAAAGATATTGG-3’) and HCO2198 (5’-TAAACTTCAGGGTGACCAAAAAATCA-3’) [42]. PCR was carried out using a 2720 Thermal Cycler (Applied Biosystems, Foster City, California, USA) with the following settings: 3 min at 94°C; followed by five cycles of 30 s at 94°C, 40 s at 45°C, and 1 min at 72°C; followed by a further 35 cycles of 30 s at 94°C, 40 s at 51°C, and 1 min at 72°C; a final extension of 10 min at 72°C and 1 min at 25°C. Successful amplification was confirmed by agarose gel electrophoresis and PCR products were subsequently cleaned by using a Nucleospin Gel and PCR Clean-up Kit (Macherey-Nagel, Düren, Germany). PCR fragments were then sequenced in both directions using the ABI Prism BigDye Terminator v3.1 Cycle Sequencing Kit (Applied Biosystems, Foster City, California, USA). Sequencing reactions were purified by ethanol precipitation, loaded on a 3500 Genetic Analyzer (Applied Biosystems, Foster City, California, USA) and analyzed with Sequencing Analysis v5.4 software. Sequences were corrected in BioEdit 7.0.9.0 [43] and primers sequences were removed from the analysis. COI sequences were translated by using the EMBOSS-Transeq website (http://www.ebi.ac.uk/Tools/emboss/transeq/index.html) to confirm the absence of nuclear pseudogenes [44]. The sequences were compared with the data in the BOLDSYSTEMS databases (http://www.barcodinglife.com/) and with the search BLASTn available on NCBI (http://blast.ncbi.nlm.nih.gov/). The sequences analyzed in this study were deposited in GenBank [accession numbers KP662045 à KP662072].

## Results

### Damage to tree species

**Fig 1:**
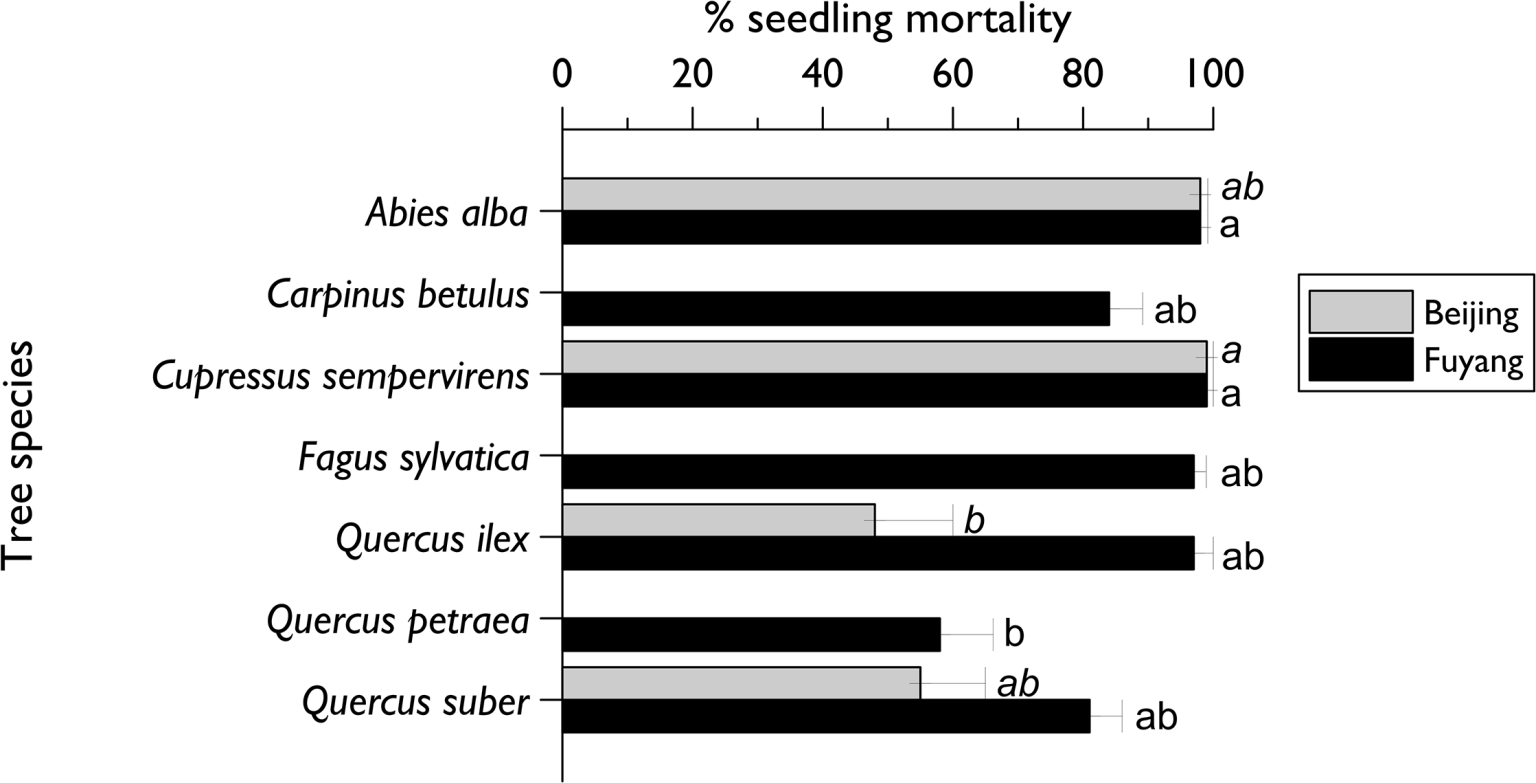
Seedling mortality per tree species observed in October 2010 at Beijing and Fuyang. Bars correspond to the mean mortality (± standard error) recorded per block of 25 seedlings. Bars from the same plot headed by the same letter (Beijing in italics, Fuyang in plain) are not significantly different (Kruskall-Wallis test followed by all-pairwise comparisons of mean ranks at α = 0.05). *Carpinus betulus*, *Fagus sylvatica* and *Quercus petraea* were not planted at the Beijing site. The Beijing plot was planted in 2007 and the Fuyang plot in 2008.

Most seedlings (ca. 95%) survived the first year following the planting. However, a high mortality then occurred at both sites with significant differences in survival between the tree species (Fig 1; Kruskall-Wallis test; Beijing: F_3, 12_ = 15.76, P< 0.001; Fuyang: F_6, 21_ = 11.12; P< 0.001). In November 2010, after three years of experiments, only 99 seedlings out of the 400 initially planted survived in Beijing. Almost all the conifers were dead (2 *Abies* and 2 *Cupressus* survived) but ca. 50% of the *Quercus* spp. (52% *Q*. *ilex* and. 45% *Q*. *suber*) were still alive. At the same date, only 86 seedlings out of 700 survived at Fuyang two years after planting, a high survival rate only occurring in *Q*. *petraea* (42 seedlings/ 100). When removed, approximately one third (34%) of the dead seedlings in Fuyang showed damage by root-feeding larvae in the genus *Holotrichia* (Scarabaeidae) and there were no differences between tree species (χ_2 = 11.0; P = 0.08_). No root-feeding larval damage was observed in Beijing, Thus, seedling death could not be attributed in most cases to insect damage but more probably to abiotic factors such as a lack of water.

**Fig 2:**
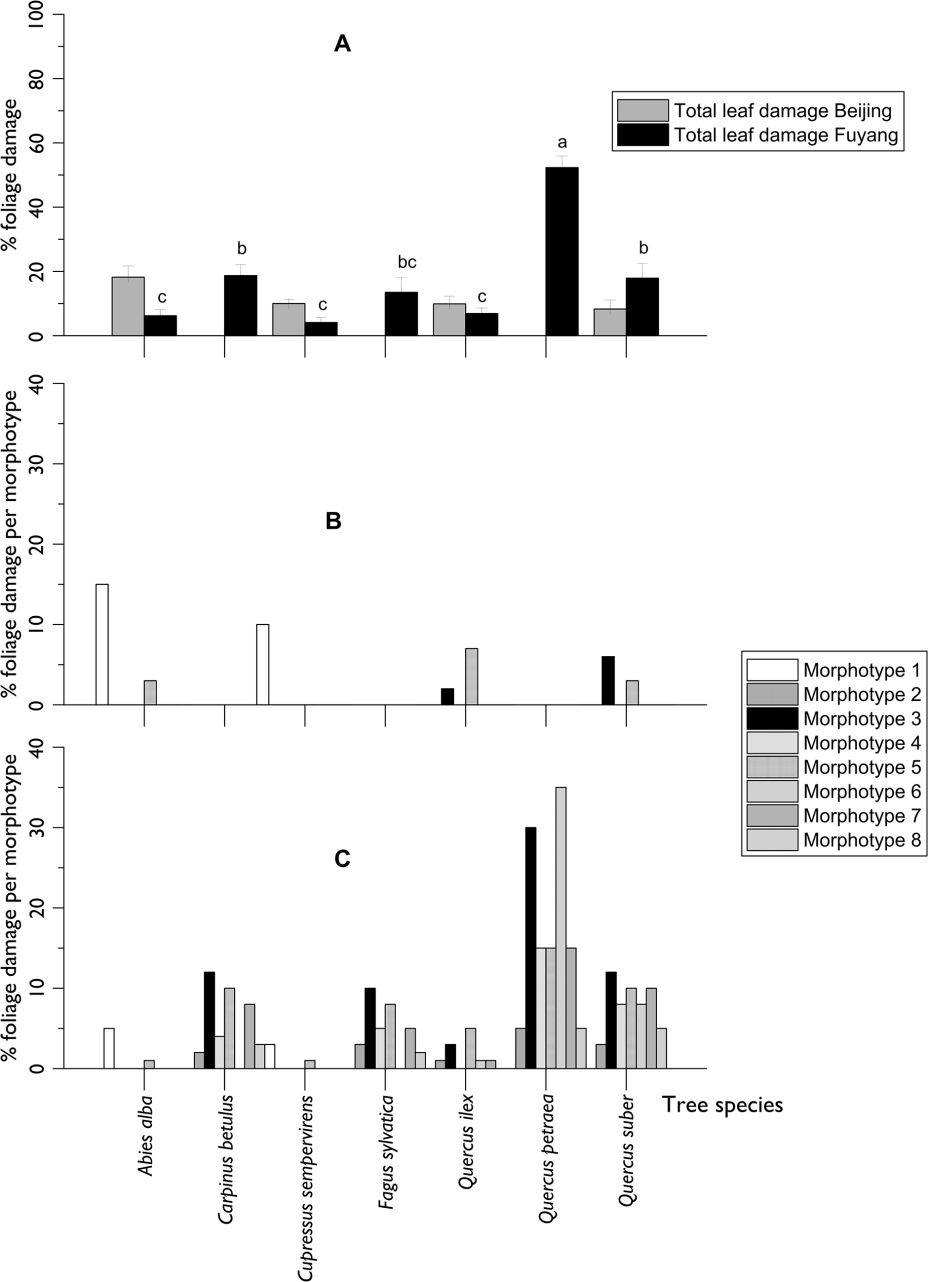
Foliage damage and damage morphotypes observed by late October 2010 at Beijing and Fuyang. A: total damage (± standard error; bars headed by the same letter are not significantly different; Kruskall-Wallis test followed by all-pairwise comparisons of mean ranks at α = 0.05); B: relative importance of morphotype damage at Beijing; C: relative importance of morphotype damage at Fuyang. In B and C, several morphotypes can be observed on the same leaf, and summation of the morphotype damage could be higher than total damage. See text for the definition of the morphotypes. The Beijing plot was planted in 2007 and the Fuyang plot in 2008.

Foliar damage by insects was classified into 8 different damage morphotypes: 1. Needles turning brown (in conifers); 2. Mines on the leaf surface, 3. The leaf surface perforated with large, irregular holes; 4. The leaf with only large veins remaining; 5. The leaf edge chewed away in an arch shape; 6. The leaf with the lower parenchyma layer eaten away; 7. The leaf with insect-made tubes hanging on the underside; and, 8. The leaf rolled up into a tube. No galls were observed. The total foliar damage measured in autumn differed considerably according to the site and tree species (Fig 2A). At Fuyang, it was significantly more important on *Quercus petraea* (>50% of the foliage on average but up to 85% on some individual seedlings) compared to other species (Fig 2A shows the values for 2010; Kruskall- Wallis test; F_6, 237_ = 19.86; P< 0.001). These species differences were stable between years (data not shown). The most frequently observed damage was the peeling away of the underside of the leaf (morphotype 6), followed by the perforation of the leaf surface with a greater or lesser skeletonization (morphotypes 3 and 4; Fig 2B). In Beijing, insect damage to the foliage was much less important (<20%) and did not differ between tree species (Fig 2A showing the values for 2010; Kruskall-Wallis test; F_3, 98_ = 1.12; P = 0.346). Only a few damage morphotypes were observed (Fig 2C). Foliage was mostly affected by damage morphotype 1 especially in *Abies alba*, which was probably caused by unidentified pathogens rather than insects.

### Insect colonization

**Fig 3:**
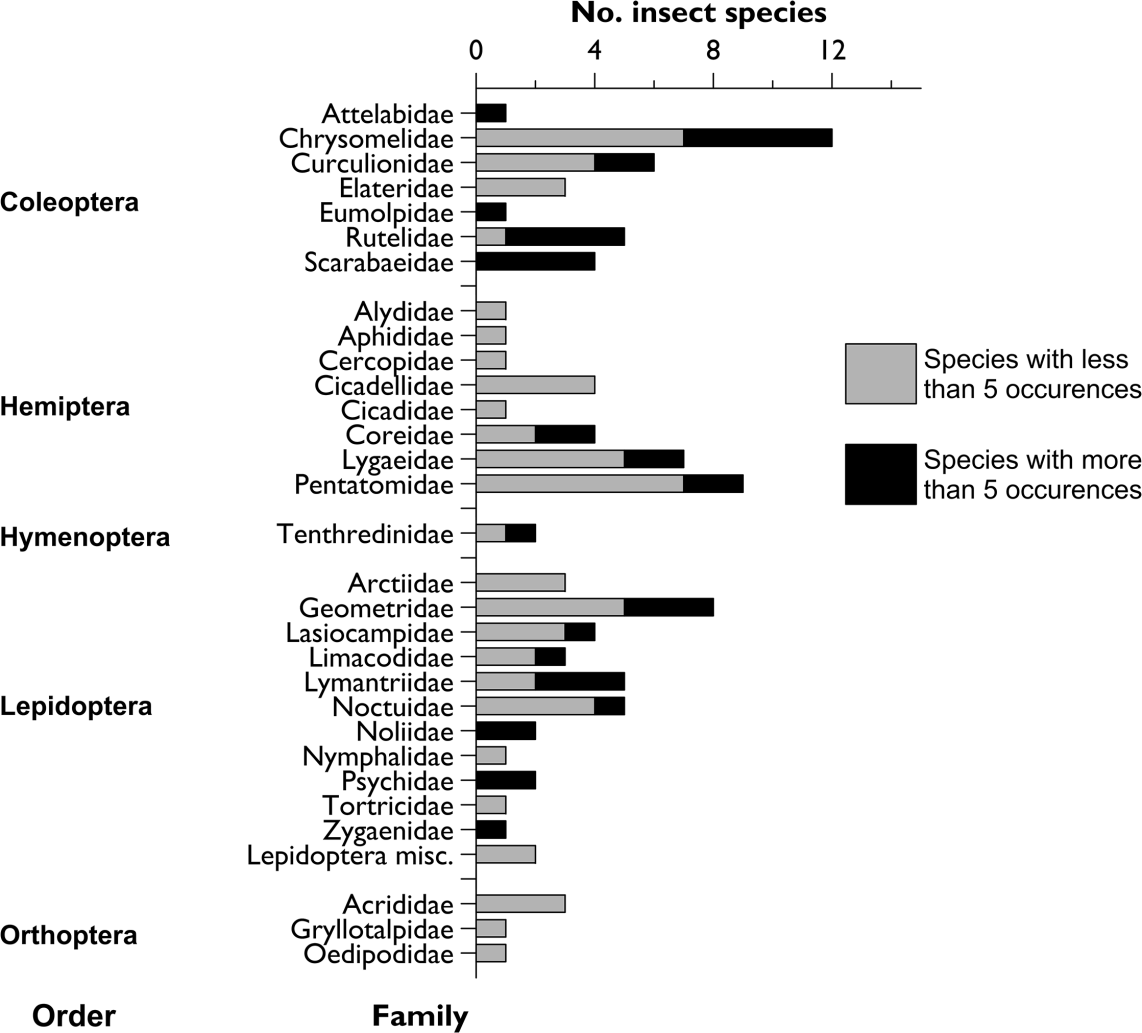
Numeric importance per family of the insect species that colonized the sentinel trees in China from 2007 to 2011. Species with more than 5 occurrences were observed on more than 5 seedlings during at least two different years. The Beijing plot was planted in 2007 and the Fuyang plot in 2008.

**Fig 4:**
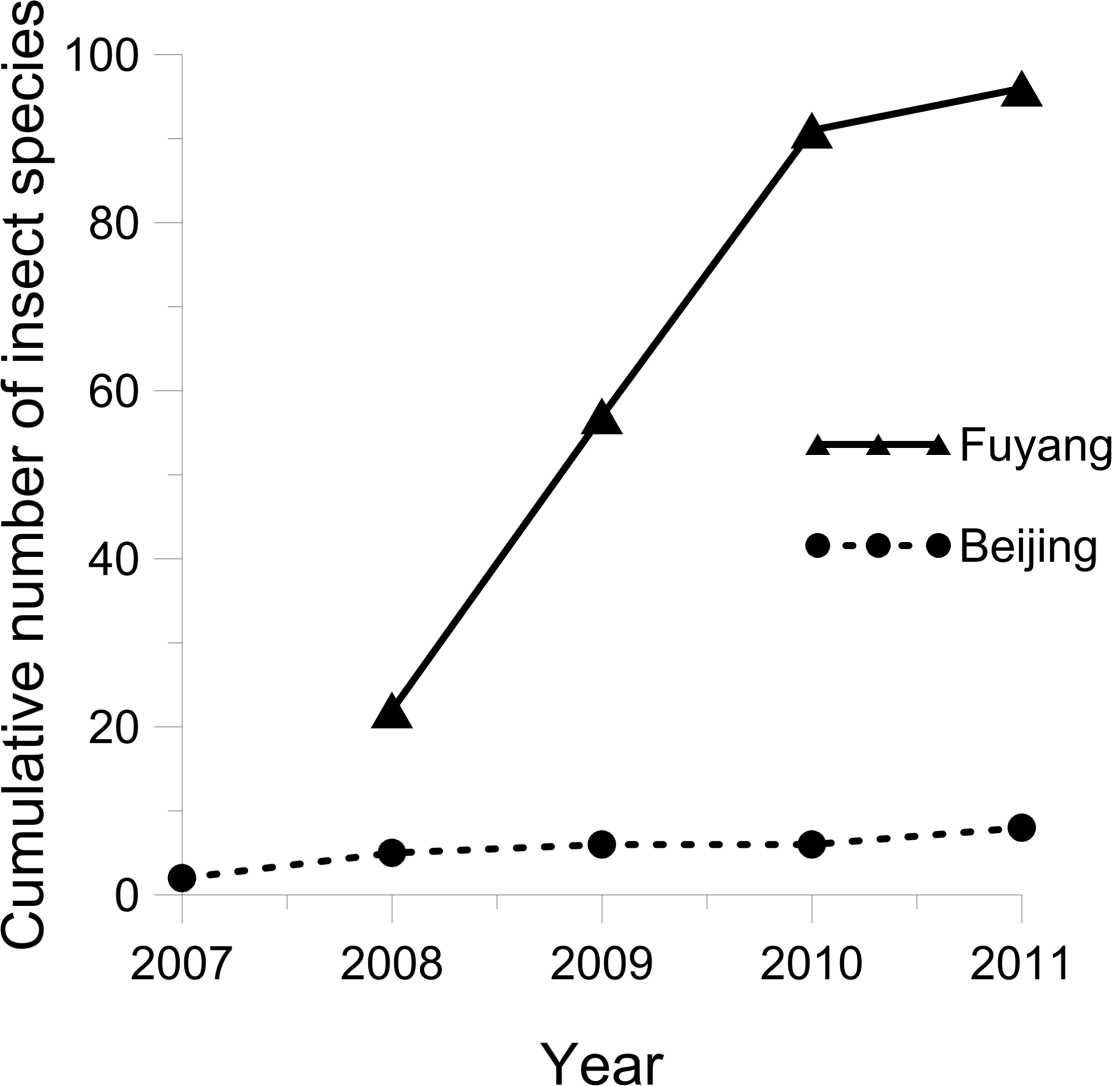
Annual changes in the cumulative number of insect species recruited by European sentinel trees planted in Beijing and Fuyang from 2007 to 2011. The Beijing plot was planted in 2007 and the Fuyang plot in 2008.

**Fig 5:**
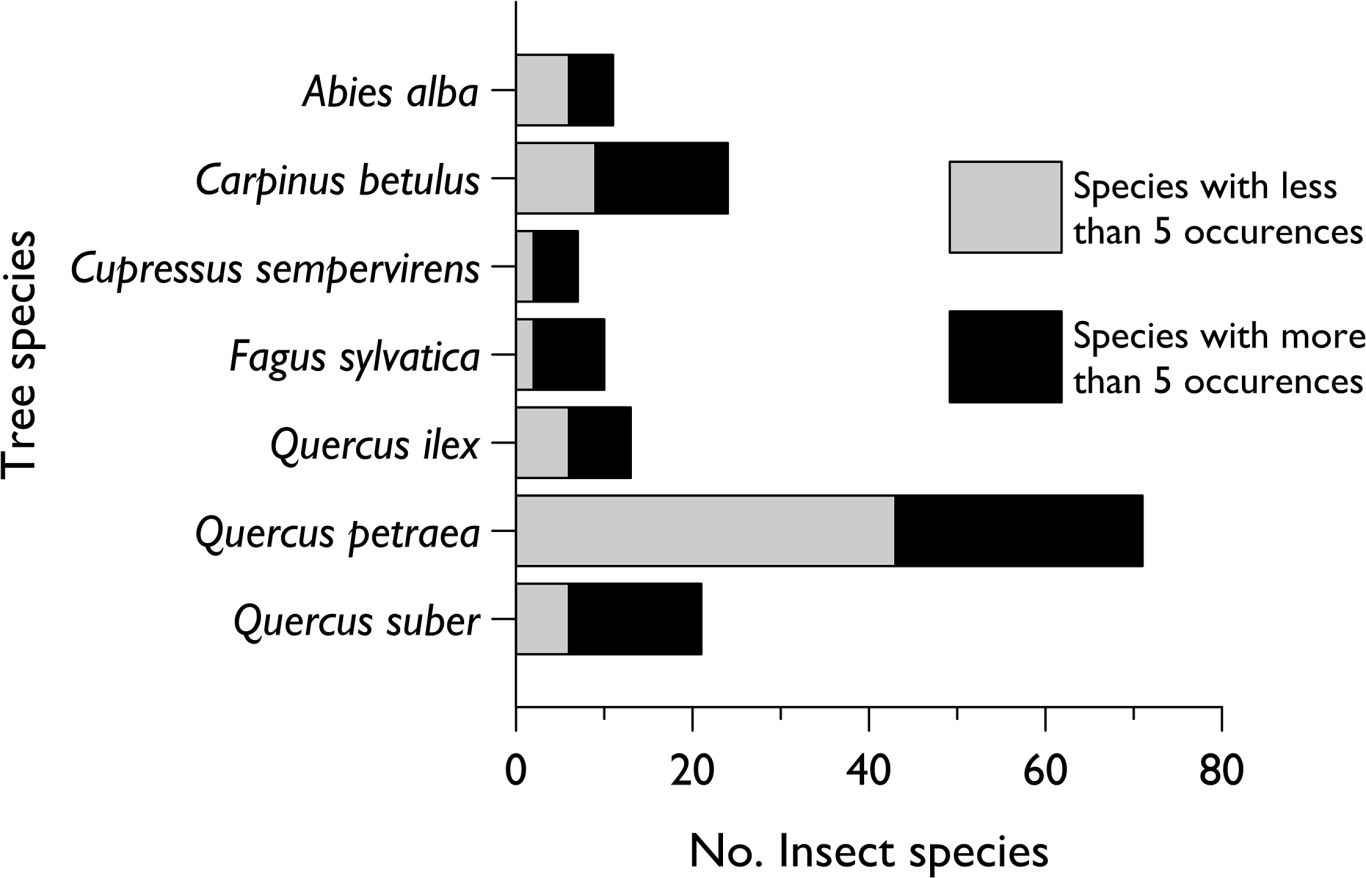
Comparative colonization of the different species of European trees planted at two sites in China and the frequency of the colonization events. No. spp > 5 events means that these insect species were observed on more than 5 seedlings during two different years (pooled over both sites). Note that an individual insect species can be observed on more than a single tree species.

A total of 596 insect specimens were collected during the whole survey (542 at Fuyang and 54 at Beijing). When the same four tree species are compared, the plants in the Fuyang trial had higher levels of colonization than in Beijing (total of 109 vs. 54 insect specimens; Student’s t test; t = -4.70; df = 3; P = 0.018). The insects belonged to a total of 104 species from 5 orders and 30 families (Fig 3; a detailed list is in S1 Table). The number of species observed in Fuyang (96 spp.) was far higher than in Beijing (8 spp.) and no species was observed at both locations. At Fuyang, the number of colonizing species was observed to increase linearly for the three first years but tended to a plateau in 2011 (Fig 4). *Quercus petraea*, which hosted 71 insect species, was colonized much more than the other tree species (Fig 5).

With 100 observed species, defoliators largely dominated the insect colonizers. Four species were root feeders as larvae but their adults were also noticed to feed on the leaves of the European plants. No xylophagous species were observed in the stems and branches. The species that colonized foliage were primarily Lepidoptera larvae (37 morphospecies in 12 different families dominated by the Geometridae, Lymantriidae and Noctuidae), adult Coleoptera (32 species in 7 different families dominated by the leaf beetles—Chrysomelidae), leaf-eating Hemiptera bugs (28 species dominated by the Pentatomidae), and sawfly larvae in the family Tenthredinidae (2 spp.). The complete list is provided in S1 Table.

Direct observations in the field together with the rearing experiments made it possible to attribute the most common damage morphotypes to particular insect groups except for morphotypes 1 and 2. Slug sawfly larvae (Hymenoptera: Tenthredinidae) were observed to peel away the bottom parenchyma layer of the leaves (morphotype 6) whilst weevils (Coleoptera: Attelabidae) created cigar-like tubes (morphotype 8). Holes on the leaf edge (morphotype 5) were mostly due to feeding by adult chafers beetles of the Rutelidae and Scarabaeidae (Coleoptera) but also to adult weevils (Coleoptera: Curculionidae). Perforations of the leaf surface (morphotype 3) were mainly made by moth larvae in the families Limacodidae, Lymantriidae and Geometridae but also by leaf beetles (Coleoptera: Chrysomelidae). Morphotype 4 occurred when leaves were so severely damaged by these moth larvae that only the thickest veins remained. Tubes hanging on the underside of the leaf corresponded to bagworm cases (Lepidoptera: Psychidae) (Morphotype 7). The larvae of 13 morphospecies were successfully reared, and six species (5 Lepidoptera and one sawfly) completed their development on the European seedlings and emerged as adults.

A total of 38 species were observed on more than 5 seedlings in at least two different years. Table 1 gives details of these colonization events including the development stage (adult, larva or pupa) in which they were observed in the plots. These species include adult Coleoptera (17 species including 5 Chrysomelidae, 4 Rutelidae and 4 Scarabaeidae), Lepidoptera larvae (14 species with 3 Geometridae and 3 Lymantriidae), Hemiptera true bugs (6 species of which 2 were Coreidae and 2 were Pentatomidae), and a sawfly hymenopteran. They were mostly observed on *Q*. *petraea* but also to a lesser extent on *Q*. *suber* and *Carpinus betulus* (Fig 5). The species with >15 observations included a slug sawfly in the genus *Caliroa*, a bagworm moth in the genus *Pteroma*, two root-feeding scarabs in the genus *Holotrichia* with adults feeding on leaves, an attelabid cigar-forming beetle in the genus *Compsapoderus*, an alticine leaf beetle, and a coreid true bug, *Rhopalus sapporensis*. Two of these species, the *Caliroa* slug sawfly and the *Pteroma* bagworm moth reached high levels of abundance, with more than 50 seedlings colonized during a year.

**Table 1:** Insect species that colonized the European sentinel trees planted in Fuyang and Beijing more than 5 times between 2007 and 2011.

Colonizing Chinese insect species					European trees planted in China							Plot sites	
Occurrence	Species	Order	Family	Known host in China	*Abies alba*	*Carpinus betulus*	*Cupressus sempervirens*	*Fagus sylvatica*	*Quercus ilex*	*Quercus petraea*	*Quercus suber*	Beijing	Fuyang
>15	*Compsapoderus continentalis* Legalov	Coleoptera	Attelabidae	?		A		A		A	A		X
>15	*Altica cirsicola* Ohno	Coleoptera	Chrysomelidae	Thistles			A				A	X	
>15	*Holotrichia diomphalia* Bates	Coleoptera	Scarabaeidae	field crops, *Azadirachta*, *Prosopis*, *Ziziphus*, *Populus*	L	L/A	L	L	L	L/A	L		X
>15	*Holotrichia trichophora* Fairm.	Coleoptera	Scarabaeidae	field crops, *Sapium*, *Cinnamomum*, *Castenea*	L	L/A	L	L	L	L/A	L		X
>15	*Rhopalus sapporensis* (Matsumura)	Hemiptera	Coreidae	Rice, field crops					A	A			X
>15	*Caliroa* sp	Hymenoptera	Tenthredinidae	Forest and fruit trees						L			X
>15	*Pteroma* nr *pendula*	Lepidoptera	Psychidae	Legume trees		L/A		L	L	L/A	L		X
>10	*Lema coronata* Baly	Coleoptera	Chrysomelidae	*Commelina communis*						A			X
>10	Geometridae sp1	Lepidoptera	Geometridae	?						L	L		X
>10	Limacodiidae sp1	Lepidoptera	Limacodiidae	?						L			X
>5	*Altica* sp	Coleoptera	Chrysomelidae	Field crops							A	X	
>5	*Lema diversa* Baly	Coleoptera	Chrysomelidae	*Commelina communis*						A			X
>5	*Nonarthra* sp.	Coleoptera	Chrysomelidae	*Beta*, *Rosa*						A			X
>5	*Calomycterus obconicus* Chao	Coleoptera	Curculionidae	Field crops, polyphagous							A		X
>5	*Echinocnemus squameus* Billberg	Coleoptera	Curculionidae	Field crops, rice						A			X
>5	*Basilepta fulvipes* (Motschulsky)	Coleoptera	Eumolpidae	*Cerasus*, *Prunus*, *Malus*, *Pterocarya*						A			X
>5	*Anisoplia* sp1	Coleoptera	Rutelidae	Field crops, fruit trees		A							X
>5	*Anisoplia* sp2	Coleoptera	Rutelidae	Field crops, fruit trees						A			X
>5	*Anomala corpulenta* Motschulsky	Coleoptera	Rutelidae	Field crops and fruit trees, *Populus*						A			X
>5	*Mimela chinensis* Kirby	Coleoptera	Rutelidae	Field crops, fruit trees		A				A			X
>5	*Holotrichia parallela* Motschulsky	Coleoptera	Scarabaeidae	*Ulmus*, *Populus*, *Salix*, field crops	L	L/A	L	L	L	L/A	L		X
>5	*Holotrichia titanus* Reitter	Coleoptera	Scarabaeidae	Field crops	L	L/A	L	L	L	L/A	L		X
>5	*Cletus tenuis* Kiritshenko	Hemiptera	Coreidae	Field crops, rice, wheat, corn		A							X
>5	*Pachybrachius* sp.	Hemiptera	Lygaeidae	field crops					A				X
>5	*Pachygrontha* sp.	Hemiptera	Lygaeidae	field crops	A								X
>5	*Dolycoris baccarum* L.	Hemiptera	Pentatomidae	field crops, fruit trees		A							X
>5	*Eysarcoris guttiger* Thunberg	Hemiptera	Pentatomidae	field crops		A				A			X
>5	Geometridae sp2	Lepidoptera	Geometridae	?				L					X
>5	*Hyposidra* sp.	Lepidoptera	Geometridae	?		L		L		L	L		X
>5	*Trabala vishnou* (Lefèbvre)	Lepidoptera	Lasiocampidae	*Juglans*, *Castanea*, *Quercus*, *Malus*						L	L		X
>5	*Cifuna* sp. nr. *locuples*	Lepidoptera	Lymantriidae	Field crops (*Lythrum salicaria*)						L	L		X
>5	*Locharna* sp	Lepidoptera	Lymantriidae	*Morus*, *Cinnamomum*, *Rosaceae*, legume trees		P				P	P		X
>5	*Olene* sp.	Lepidoptera	Lymantriidae	*Pauwlonia*, *Populus*, *Salix*, *Malus*						L			X
>5	*Acronicta rumicis* (L.)	Lepidoptera	Noctuidae	*Pyrus*, *Malus*, *Amygdalus*, *Rumex*, *Polygonum*						L			X
>5	*Nola* sp	Lepidoptera	Nolidae	*Punica*						L	L		X
>5	*Manoba* sp	Lepidoptera	Nolidae	*Punica*						L			X
>5	Psychidae sp	Lepidoptera	Psychidae	?		L							X
>5	Zygaenidae sp	Lepidoptera	Zygaeneidae	?		L				L			X

Insect stage: A: Adult; L: larva; P: pupae; E: eggs

### Insect identification

Only a small proportion of the insects collected could be directly identified to species using classical taxonomy (22 out of 104 species; i.e. 21.1%). The sequencing of morphospecies using the ‘barcode’ gene suggested a specific identity for 5 more organisms with a match of at least 99% in the barcode database. Their identification was then confirmed from the relevant literature. The sequencing of all the lepidopteran larvae and pupae provided 5 species identities (match >99%), 13 genus identities (match between 98% and 97%), and 18 family identities (match >95%). However, the families of 2 Lepidoptera larvae were unknown (match <90%). In total, 27 of the 104 morphospecies were identified to the species (26.0%), and 25 to the genus level (24.0%). These percentages were higher in the species that had more than 5 colonization events, with 19 identified to species (50.0%) and 14 to the genus level (36.8%; Table 1).

Of the species identified, only a few were found to have originated with certainty from forest trees or woody plants (6 spp.), and none from direct relatives of European trees (e.g. Fagaceae for *Quercus* spp.). However, the large number of specimens unidentified to the species level prevents any definite conclusions. Of the 38 species with more than 5 colonization events, at least 26 appeared to be either polyphagous or to have switched from agricultural crops or fruit trees (Table 1).

## Discussion

Our sentinel tree plantings in China supplied a new list of Asian insects potentially threatening European trees. Almost all of these species have never been intercepted by the European quarantine services, nor have they previously been considered as potential threats for introduction to other continents because reports of damage have been limited, or sometimes unknown in their native Chinese range. Although at least six species were shown to be capable of developing to adults when reared on the European seedlings, neither the ability to complete larval development on these new hosts, nor the consequences for their subsequent reproductive success could not be systematically tested for all insect species. Caution has therefore to be taken when evaluating the results. Although a number of the observed insects were probably just incidental feeders on the European seedlings, the 38 species with more than five colonization events could be considered as being capable of switching to European trees if introduced to Europe. Of these 38 insect species, seven were frequently observed (more than 15 records) on the seedlings.

These seven species include two species of white grubs in the genus *Holotrichia*, whose larvae feed on roots and are capable of killing seedlings. Heavy damage by *Holotrichia* spp. has been reported on groundnuts as well as other commercial crops such as potato, pea and maize in India [45] but also on bamboos (*Bambusa* spp.) [46]. The larvae (bagworms) of a species of Psychid moth, *Pteroma* nr. *pendula*, were capable of defoliating the seedlings of all the tested broadleaved trees. Species in this genus are usually polyphagous on a wide range of legume trees and palms [47], and outbreaks of *Pteroma* have been reported in Malaysia on *Acacia mangium* and oil palm, *Elaeis guineensis* [48]. Similarly, the larvae of a tenthredinid slug sawfly, *Caliroa* sp., peeled away most of the leaf undersurface of a large number of seedlings of *Q*. *petraea*. Species in the genus *Caliroa*, e.g. *C*. *matsumotonis*, are usually found on fruit trees such plum, pear and peach [49]. A leaf-rolling weevil, *Compsapoderus continentalis*, was very abundant and caused considerable defoliation each year, especially on *Q*. *petraea* but also on *Q*. *suber*, *F*. *sylvatica* and *C*. *betulus*. It has only been recently described from China and Korea [50] and no data are available on its biology and host plants as yet. The adults of the alticine leaf beetle *Altica cirsicola*, which was frequently observed in Beijing on *Q*. *suber* and *C*. *sempervirens*, are known to develop on thistles, *Cirsium* spp. [51]. The seventh species, a coreid true bug, *Rhopalus sapporensis* (syn. *Aeschyntelus sparsus)*, whose adults fed regularly on leaves of *Quercus* spp has been previously recorded as a pest of rice in Korea [52] and of tobacco in China [53]. Such species would have probably not appeared using the different types of clustering methods proposed to identify species at risk [29, 32] because these methods rely on distribution data for known invasive species and major pests whereas most insects observed in the experiment were not previously considered as pests in China nor invasive elsewhere.

The likelihood of introduction to Europe is however considerably different between the seven insect species. *Holotrichia* grubs are unlikely to be introduced because the plant trade is usually based on bare rooted seedlings without soil, and their large adults are very conspicuous. In contrast, *Pteroma* bagworms and *Caliroa* slug sawflies, which may overwinter inconspicuously as eggs on twigs and branches, constitute real threats if we take into account their capability for defoliation. A sawfly recently introduced from Eastern Asia, *Aproceros leucopoda*, has spread all over Central Europe in a few years since its first observation in 2003 and it is seriously defoliating elm (*Ulmus* spp.) stands [54]. Preliminary host screening tests under quarantine conditions in France also confirmed that *Pteroma* nr. *pendula* can easily develop on the foliage of most European broadleaved trees, e.g. *Quercus*, *Acer* and *Populus*, with several generations per year causing a rapid and severe defoliation of the plants tested (O. Denux, unpublished observations).

There was no overlap in the insect fauna observed on the European seedlings between the two plantations, although this was not surprising given the distance between them and the different environments in which they were set up. The limited colonization of the Beijing plot, which was planted within a nursery production site, was somewhat reassuring because it may indicate that phytosanitary measures applied in the surrounding plots could have affected pest presence, and thus have limited the pool of pest specimens that would be candidates for transportation by the nursery plant trade. By contrast, judging by the species identifications that have been made, the extensive colonization of the Fuyang plot appeared to have mostly been caused by pests of agricultural crops rather than forest trees even though the plot was set up in a forested environment. The absence in the vicinity of native tree species congeneric to the European ones may be an explanation since it is well known that alien plants that have congeneric natives in the region they are introduced tend to recruit herbivores more rapidly than those without congeneric natives, the herbivores mostly switching from the congeneric natives [38, 55, 56]. In addition, this pattern is likely to be related to the high degree of polyphagy exhibited by a number of crop pests attracted to the European trees in Fuyang.

Although the potential to use sentinel plants to identify possible insect threats is promising, several problems with this method must be pointed out. A major one is the difficulty of taxonomic identification since a large proportion of the collected specimens are larvae for which identification keys do not exist in most groups. Rearing larvae to adults is usually complicated because of limitations in food availability, particularly because the sentinel plants need to be preserved. In addition, some species could still be unknown to science; e.g. the weevil *Compsapoderus continentalis* was only described in 2007 [50]. A systematic molecular analysis of all collected specimens could also be used in taxonomic identification of immature stages given that data from established barcodes datasets are steadily increasing and becoming more accepted by biosecurity agencies [57]. However, such molecular analyses revealed considerable limitations in our work because the development of reference molecular databases is, as yet, unequal between taxonomic groups. DNA barcodes enabled identifications to the species level with certainty for only a few moth larvae. It was generally sufficient to identify moth and sawfly specimens to genus but not for all true bugs and leaf beetles.

All except four root-damaging species were foliage feeders. This is directly related to the design of the experiment that only studied seedlings up to the 5^th^ year of growth at most. Thus, unless the plantations are maintained long enough to obtain mature trees, these sentinel trees are mainly suitable for screening pests of seedlings and young trees, which mostly consist of foliar or root-feeding organisms. Thus, the method is not appropriate for bark and wood borers or fruit/cone pests which develop on larger trees.

The precise way in which seedlings recover following translocation is of major concern because it is still not understood whether the insect attraction to sentinel trees was related to stress following planting. In fact, seedling mortality began to show up the year after translocations but remained limited at that time and a significant mortality only began to be observed during the second year of the experiment. The reasons for this high mortality could not be determined with certainty, and may be due to abiotic factors. The important and repeated foliar damage, by both insects and pathogens on *Quercus petraea* at Fuyang, as well as pathogen damage on *Abies alba* at Beijing, probably affected seedling growth and survival but cannot be responsible for the high mortality observed in species such as *Cupressus sempervirens* and *Q*. *ilex*, which had little defoliation at both sites. Climatic factors are likely to be involved in Beijing due to the unusually severe cold and snowy winter of 2010 but winter mortality also occurred at Fuyang. However, the conditions of importation in Fuyang constituted a major problem because the seedlings had been stored without watering for several weeks under quarantine conditions before being planted. It is likely that these conditions have seriously affected the acclimatization and survival of the seedlings in the new environment.

Another major problem with the sentinel tree method is logistical. Authorizations for planting European trees in China were very difficult to obtain and administrative hurdles delayed the first plantation in such a way that many trees died and new shipments and plantations had to be organized. Although the areas for the sentinel plantations were primarily defined by climate matching with European regions, it proved impossible to plant the sentinels as planned because of administrative reasons. Indeed, importations of such exotic plants are already forbidden in many countries and will increasingly be banned in the future. Therefore, new plantations are likely to be made with seedlings grown from seeds in the country of planting and this will further increase the duration of the project. The planting of sentinel trees thus requires both long-term funding, strong local links and reliable collaborators.

An alternative method is based on arboretum surveys, which can rely on a worldwide network of arboreta, botanical gardens and nurseries where a large number of foreign plants are planted in continents different from those where they are native [37, 39]. Groenteman et al. [58] thus sampled New Zealand tree species planted in botanical gardens and arboreta of California in order to check their possible colonization by invasive Hemiptera and their associated phytopathogenic bacteria, both of which are not yet present in New Zealand. The International Sentinel Plant Network (ISPN- http://www.plantsentinel.org) linked to Botanic Gardens Conservation International (www.bgci.org) is currently developing this method, which has already been successfully applied to the study of pathogens colonizing European host trees planted in Siberia [39]. Logistically, it is easier to organize since it relies on managers of established botanical gardens. It may also allow surveys of the recruitment of insects by mature trees, and especially of groups like xylophagous pests which could not be monitored thoroughly by our sentinel plantations which used small seedlings for a relatively short period of time. However, a major advantage of the sentinel tree method compared to arboretum surveys is the use of high numbers of individual trees of the same species, which enables statistical analyses of pest damage to be made without having to take into account the genetic variability of tree species because arboreta usually host only a few individuals of each tree species, with a limited genetic variability. In conclusion, both methods can be considered as complementary.

## Supporting Information

S1 Table List of the 104 insect species that colonized the European sentinel trees planted in Fuyang and Beijing between 2007 and 2011. Insect stage: A: Adult; L: larva; P: pupae; E: eggs.
(DOCX)
