# Supplementary material for: Planting Sentinel European Trees in Eastern Asia as a Novel Method to Identify Potential Insect Pest Invaders
Source: PLoS One. 2015 May 20;10(5):e0120864. doi: 10.1371/journal.pone.0120864 (PMC4439023; doi:10.1371/journal.pone.0120864)
Supplement: S1 Table — Insect stage: A: Adult; L: larva; P: pupae; E: eggs. (DOCX) [file pone.0120864.s001.docx]

| Colonizing Chinese insect species | | | | Plot sites | | European trees planted in China | | | | | | |
| --- | --- | --- | --- | --- | --- | --- | --- | --- | --- | --- | --- | --- |
| Species | Order | Family | Occurrence | Fuyang | Beijing | *Abies alba* | *Carpinus betulus* | *Cupressus sempervirens* | *Fagus sylvatica* | *Quercus ilex* | *Quercus petraea* | *Quercus suber* |
| *Compsapoderus continentalis* Legalov | Coleoptera | Attelabidae | >15 | X |  |  | A |  | A |  | A | A |
| *Altica cirsicola* Ohno | Coleoptera | Chrysomelidae | >15 |  | X |  |  | A |  |  |  | A |
| *Altica* sp. | Coleoptera | Chrysomelidae | >5 |  | X |  |  |  |  |  |  | A |
| *Cryptocephalus* sp. | Coleoptera | Chrysomelidae | <5 | X |  |  |  |  | A |  |  |  |
| *Lema coronata* Baly | Coleoptera | Chrysomelidae | >10 | X |  |  |  |  |  |  | A |  |
| *Lema diversa* Baly | Coleoptera | Chrysomelidae | >5 | X |  |  |  |  |  |  | A |  |
| *Nonarthra* sp. | Coleoptera | Chrysomelidae | >5 | X |  |  |  |  |  |  | A |  |
| *Phaedon* sp. | Coleoptera | Chrysomelidae | <5 | X |  | A |  |  |  |  | A |  |
| Chrysomelidae (Galerucinae) sp1 | Coleoptera | Chrysomelidae | <5 | X |  |  |  |  |  |  | A |  |
| Chrysomelidae (Galerucinae) sp2 | Coleoptera | Chrysomelidae | <5 | X |  |  | A |  |  |  | A |  |
| Chrysomelidae sp3 | Coleoptera | Chrysomelidae | <5 | X |  |  |  |  |  |  | A |  |
| Chrysomelidae sp4 | Coleoptera | Chrysomelidae | <5 | X |  |  |  |  |  |  | A |  |
| Chrysomelidae sp5 | Coleoptera | Chrysomelidae | <5 | X |  |  |  |  |  |  | A |  |
| *Apion* sp. | Coleoptera | Curculionidae | <5 | X |  |  |  |  |  |  | A |  |
| *Calomycterus obconicus* Chao | Coleoptera | Curculionidae | >5 | X |  |  |  |  |  |  |  | A |
| *Echinocnemus squameus* Billberg | Coleoptera | Curculionidae | >5 | X |  |  |  |  |  |  | A |  |
| Curculionidae sp1 | Coleoptera | Curculionidae | <5 | X |  |  |  |  |  |  | A |  |
| Curculionidae sp2 | Coleoptera | Curculionidae | <5 | X |  |  | L |  |  |  |  |  |
| Curculionidae sp3 | Coleoptera | Curculionidae | <5 | X |  |  |  |  |  |  | A |  |
| Elateridae sp1 | Coleoptera | Elateridae | <5 | X |  |  |  |  |  |  | A |  |
| Elateridae sp2 | Coleoptera | Elateridae | <5 | X |  |  |  | A |  |  |  |  |
| Elateridae sp3 | Coleoptera | Elateridae | <5 | X |  |  |  | A |  |  |  |  |
| *Basilepta fulvipes* (Motschulsky*)* | Coleoptera | Eumolpidae | >5 | X |  |  |  |  |  |  | A |  |
| *Anisoplia* sp1 | Coleoptera | Rutelidae | >5 | X |  |  | A |  |  |  |  |  |
| *Anisoplia* sp2 | Coleoptera | Rutelidae | >5 | X |  |  |  |  |  |  | A |  |
| *Anomala* *corpulenta* Motschulsky | Coleoptera | Rutelidae | >5 | X |  |  |  |  |  |  | A |  |
| *Mimela chinensis* Kirby | Coleoptera | Rutelidae | >5 | X |  |  | A |  |  |  | A |  |
| *Mimela holosericea* Kirby | Coleoptera | Rutelidae | <5 | X |  |  |  |  |  |  | A |  |
| *Holotrichia diomphalia* Bates | Coleoptera | Scarabaeidae | >15 | X |  | L | L/A | L | L | L | L/A | L |
| *Holotrichia* *parallela Motschulsky* | Coleoptera | Scarabaeidae | >5 | X |  | L | L/A | L | L | L | L/A | L |
| *Holotrichia titanus* Reitter | Coleoptera | Scarabaeidae | >5 | X |  | L | L/A | L | L | L | L/A | L |
| *Holotrichia* *trichophora* Fairm. | Coleoptera | Scarabaeidae | >15 | X |  | L | L/A | L | L | L | L/A | L |
| *Riptortus pedestris* (F.) | Hemiptera | Alydidae | <5 | X |  | A |  |  |  |  |  |  |
| *Aphis* sp. | Hemiptera | Aphididae | <5 |  | x |  |  |  |  | A/L |  |  |
| Cercopidae sp. | Hemiptera | Cercopidae | <5 | X |  |  |  |  |  |  | L |  |
| *Cicadella viridis* (L.) | Hemiptera | Cicadellidae | <5 |  | X |  |  |  |  | A |  | A |
| Cicadellidae sp1 | Hemiptera | Cicadellidae | <5 | X |  |  |  |  |  |  | A |  |
| Cicadellidae sp2 | Hemiptera | Cicadellidae | <5 | X |  |  |  |  |  |  | A |  |
| Cicadellidae sp3 | Hemiptera | Cicadellidae | <5 | X |  |  |  |  |  |  | A |  |
| Cicadidae sp. | Hemiptera | Cicadidae | <5 | X |  |  |  |  |  |  | A |  |
| *Cletus tenuis* Kiritshenko | Hemiptera | Coreidae | >5 | X |  |  | A |  |  |  |  |  |
| Coreidae sp1 | Hemiptera | Coreidae | <5 | X |  |  | A |  |  |  |  |  |
| Coreidae sp2 | Hemiptera | Coreidae | <5 | X |  |  | A |  |  |  |  |  |
| *Orsillus* sp. | Hemiptera | Lygaeidae | <5 | X |  |  | A |  |  |  |  |  |
| *Pachybrachius* sp. | Hemiptera | Lygaeidae | >5 | X |  |  |  |  |  | A |  |  |
| *Pachygrontha* sp. | Hemiptera | Lygaeidae | >5 | X |  | A |  |  |  |  |  |  |
| Lygaeidae sp1 | Hemiptera | Lygaeidae | <5 | X |  |  |  |  |  |  | A |  |
| Lygaeidae sp2 | Hemiptera | Lygaeidae | <5 | X |  | A |  |  |  |  |  |  |
| Lygaeidae sp3 | Hemiptera | Lygaeidae | <5 | X |  |  |  |  |  |  | A |  |
| Lygaeidae sp4 | Hemiptera | Lygaeidae | <5 | X |  |  |  |  |  | A |  |  |
| *Aeschyntelus sparsus* Blote | Hemiptera | Pentatomidae | >15 | X |  |  |  |  |  | A | A |  |
| *Dolycoris baccarum* L. | Hemiptera | Pentatomidae | >5 | X |  |  | A |  |  |  |  |  |
| *Eurydema dominulus* (Derjanschi & Péricart) | Hemiptera | Pentatomidae | <5 |  | X |  |  |  |  | A |  | A |
| *Eysarcoris guttiger* Thunberg | Hemiptera | Pentatomidae | >5 | X |  |  | A |  |  |  | A |  |
| *Picromerus* sp. | Hemiptera | Pentatomidae | <5 | X |  |  | E |  |  |  | E |  |
| Pentatomidae sp1 | Hemiptera | Pentatomidae | <5 |  | X | A |  |  |  |  |  |  |
| Pentatomidae sp2 | Hemiptera | Pentatomidae | <5 | X |  |  |  |  |  |  | A |  |
| Pentatomidae sp3 | Hemiptera | Pentatomidae | <5 | X |  |  |  |  |  |  | A |  |
| Pentatomidae sp4 | Hemiptera | Pentatomidae | <5 | X |  |  |  |  |  |  | A |  |
| Pentatomidae sp5 | Hemiptera | Pentatomidae | <5 | X |  |  |  |  |  |  | A |  |
| *Caliroa* sp. | Hymenoptera | Tenthredinidae | >15 | X |  |  |  |  |  |  | L |  |
| Tenthredinidae sp. | Hymenoptera | Tenthredinidae | <5 | X |  |  | P |  |  |  |  |  |
| *Cheliosea* sp. | Lepidoptera | Arctiidae | <5 | X |  |  |  |  |  |  | L |  |
| *Eilema* sp. | Lepidoptera | Arctiidae | <5 | X |  |  |  |  |  |  | L |  |
| *Spilarctia* sp. | Lepidoptera | Arctiidae | <5 | X |  |  |  |  |  |  | L |  |
| *Hyposidra* sp. | Lepidoptera | Geometridae | >5 | X |  |  | L |  | L |  | L | L |
| *Scopula* sp. | Lepidoptera | Geometridae | <5 | X |  |  | P |  |  |  |  |  |
| Geometridae sp1 | Lepidoptera | Geometridae | >10 | X |  |  |  |  |  |  | L | L |
| Geometridae sp2 | Lepidoptera | Geometridae | >5 | X |  |  |  |  | L |  |  |  |
| Geometridae sp3 | Lepidoptera | Geometridae | <5 | X |  |  |  |  |  |  | L |  |
| Geometridae sp4 | Lepidoptera | Geometridae | <5 | X |  |  |  |  |  |  | L |  |
| Geometridae sp5 | Lepidoptera | Geometridae | <5 | X |  |  |  |  |  |  | L |  |
| Geometridae sp6 | Lepidoptera | Geometridae | <5 | X |  |  |  |  |  |  | L |  |
| *Trabala vishnou* (Lefèbvre) | Lepidoptera | Lasiocampidae | >5 | X |  |  |  |  |  |  | L | L |
| *Trabala* sp. | Lepidoptera | Lasiocampidae | <5 | X |  |  |  |  |  |  |  | L |
| Lasiocampidae sp1 | Lepidoptera | Lasiocampidae | <5 | X |  |  |  |  |  |  |  | P |
| Lasiocampidae sp2 | Lepidoptera | Lasiocampidae | <5 |  | X |  |  |  |  | L |  |  |
| Limacodidae sp1 | Lepidoptera | Limacodidae | >10 | X |  |  |  |  |  |  | L |  |
| Limacodidae sp2 | Lepidoptera | Limacodidae | <5 | X |  |  |  |  |  |  | L |  |
| Limacodidae sp3 | Lepidoptera | Limacodidae | <5 | X |  |  |  |  |  |  | L |  |
| *Calliteara grotei* (Moore) | Lepidoptera | Lymantriidae | <5 | X |  |  |  |  |  |  | L |  |
| *Cifuna* sp. nr. *locuples* | Lepidoptera | Lymantriidae | >5 | X |  |  |  |  |  |  | L | L |
| *Locharna* sp | Lepidoptera | Lymantriidae | >5 | X |  |  | P |  |  |  | P | P |
| *Olene* sp. | Lepidoptera | Lymantriidae | >5 | X |  |  |  |  |  |  | L |  |
| Lymantriidae sp | Lepidoptera | Lymantriidae | <5 | X |  |  |  |  |  |  | L |  |
| *Acronicta rumicis* (L.) | Lepidoptera | Noctuidae | >5 | X |  |  |  |  |  |  | L |  |
| *Acronicta* sp. | Lepidoptera | Noctuidae | <5 | X |  |  |  |  |  |  | L | L |
| *Anomis mesogona* Walker | Lepidoptera | Noctuidae | <5 | X |  |  |  |  |  |  | L |  |
| Noctuidae sp1 | Lepidoptera | Noctuidae | <5 | X |  |  |  |  |  |  | L |  |
| Noctuidae sp2 | Lepidoptera | Noctuidae | <5 | X |  |  |  |  |  |  | L |  |
| *Nola* sp | Lepidoptera | Nolidae | >5 | X |  |  |  |  |  |  | L | L |
| *Manoba* sp | Lepidoptera | Nolidae | >5 | X |  |  |  |  |  |  | L |  |
| *Acraea issoria* Hübner | Lepidoptera | Nymphalidae | <5 | X |  | L |  |  |  |  |  |  |
| *Pteroma* nr *pendula* | Lepidoptera | Psychiidae | >15 | X |  |  | L/A |  | L | L | L/A | L |
| Psychiidae sp | Lepidoptera | Psychiidae | >5 | X |  |  | L |  |  |  |  |  |
| Tortricidae sp | Lepidoptera | Tortricidae | <5 |  | X |  |  |  |  |  |  | L |
| Zygaenidae sp | Lepidoptera | Zygaeneidae | >5 | X |  |  | L |  |  |  | L |  |
| Lepidoptera sp1 | Lepidoptera | ? | <5 | X |  |  |  |  |  |  | L |  |
| Lepidoptera sp2 | Lepidoptera | ? | <5 | X |  |  |  |  |  |  | L |  |
| *Xenocatantops brachycerus* (Will) | Orthoptera | Acrididae | <5 | X |  |  |  |  | A |  |  |  |
| Orthoptera sp1 | Orthoptera | Acrididae | <5 | X |  |  |  |  |  |  | L |  |
| Orthoptera sp2 | Orthoptera | Acrididae | <5 | X |  |  | L |  |  |  | L |  |
| *Gryllotalpa* sp. | Orthoptera | Gryllotalpidae | <5 | X |  |  |  |  |  | A | A |  |
| Orthoptera sp3 | Orthoptera | Oedipodidae | <5 | X |  | L |  |  |  |  |  |  |
